# Annotations

**Published:** 1888-08-11

**Authors:** 


### Annotations.

**Pity the Poor Practitioner.** Now that the Parliamentary Session is moribund, and the dead—or, as some people call it, the silly—season is coming on, we offer a suggestion to the newspapers and those who like to write letters to the editor. Let them take up as the subject of their lucubrations, "What shall we do with our medical students?" who at this season are cast upon the world in hundreds as newly-capped practitioners, bearing proudly their hardly won diplomas. It seems that a fair proportion of them seem destined to swell the ranks of the unemployed, if we may judge from two advertisements cut from a London daily paper, and forwarded by a correspondent to the *British Medical Journal*. One tells the pitiful tale that "a young Medical Man, of great abilities and high testimonials, is, from want of means, prevented from obtaining a practice," and appeals to the benevolent to help him by getting him an appointment "or otherwise"—otherwise probably meaning money down. The second advertiser starts off with the heading: "To Philanthropists," and offers to attend gratis a limited number of patients recommended by a philanthropist who will pay his rent and taxes. On the whole we like him better than the other, for he offers at once to work off his debt; but both cases display a sad state of affairs. It seems obvious, even if we surmise that the abilities of these gentlemen are not quite so great as they profess, that at present we have either too many doctors or too few patients. What causes this? In the first place an injudicious rush into an over-crowded profession of those who forget that to push one's way to the front, or any way near it, requires time, and time means money, for the young doctor must have, at least, a limited amount of food, and must—alas for him! "keep up an appearance." Some of our successful doctors could tell sad tales of early privations if they chose; how many perish in the struggle, or are forced to give up the high ambitions they cherished and take subordinate places, "God only and good angels know." But are not the patients to blame if they take medical relief at a hospital when they can afford to pay a private practitioner? Are not the hospitals to blame if they receive patients without inquiring into their means! Nay, are not our great doctors to blame if, to gratify the most amiable emotions, they give gratuitous treatment to others than the really poor? The doctrine that "true charity is an intelligent selfishness" is capable of much misinterpretation, but it is none the less one that contains much truth.

**Child-Cheats.** A certain doctor owned a dog named Jim—most intelligent and amiable of the bull-dog tribe!—who, in an amicable trial of strength with a friend, broke one of his fore-legs. His master set the limb, and Jim recovered, though his unfortunate habit of gnawing his bandages and tearing them off caused the cure to be less perfect than was desirable, and to the end of his days he was distinguished by a slight, and not ungraceful limp. After the accident, if Jim was guilty of any canine sin he would receive the severe remonstrance his fault deserved, and sometimes a threat of punishment. It never got beyond a threat, for when he saw his master go to the drawer where the whip was kept, he would hobble-up to him with such an exaggerated limp that no one but a villain of the deepest dye could have struck a dog who apparently was suffering so much. Finally he would put the paw of the wounded leg on his master's knee, and look at him with such an expression of agony that the incident always ended with the remark, "Jim, you're a sly old malingerer," followed by forgiveness and caresses, which caused Jim's excessive lameness to disappear with miraculous rapidity. Yet dogs are not, on the whole, deceitful animals, but get credit for many superhuman virtues. Neither are children deceitful, but it is to be feared that occasionally they will, like Jim, assume a disease when they have it not, especially if thereby they can escape some disagreeable task. So common has this become, and so often have doctors been deceived by these little cheats, that Dr. L. Dufestel has recently published a monograph on "The Simulated Diseases of Childhood" (Thèse de Paris, 1888). He divides these youthful deceivers into three classes. First, those who feign some simple indisposition with a definite object, and who are otherwise normally constituted; second, children who pretend to be suffering from more complicated diseases, who present, usually, an hereditary history of nervous affections, and who are not well balanced mentally; and third, hysterical malingerers. The second class is the most difficult to deal with, as the patients are of that nervous constitution which absolutely feels every symptom described, though a sufficient cause may be absent, and in many cases they have been badly brought up, their parents having believed they were delicate, and so yielded to their every whim. We regret to say that Dr. Dufresnel's statistics show two-thirds of these young malingerers to be girls, and he prudently advises that the harsh treatment the physician feels inclined to exhibit on meeting with such cases should not be tried till the child-cheat's parents are convinced that the disease is imaginary, if not wilfully pretended.

**The Insurance of Children.** "Those pests of society—those deadly societies which insure children—those societies which seem to be instituted for the destruction of children, for the perpetration of murder." These are strong words, but there can be little doubt that Mr. Justice Day, when he uttered them at Salisbury Assizes last month, was justified in every expression he used. He was trying the case of a man named George Hayes and his wife, who were accused of starving and otherwise neglecting some children placed in their charge. For all these children they had received premiums, varying in amount, from parents who, for one sad reason or another, avoided the responsibility of bringing up their offspring themselves; and all the children were insured in what was misnamed a friendly society—no friend to the children—which would pay five or six pounds in the event of the death of any one of them. That is, Mrs. Hayes and her husband had got all they could out of the children's lives, but their deaths would be profitable, therefore they were to be allowed to die. One of them, Zoe Graham, a child of two years, was suffering from "rickets," the softening of all the bones in the body from lack of proper food. The normal weight of a child of that age is 24 lbs.; this child weighed only 10 lbs. Had not the Society for Preventing Cruelty to Children heard of Mrs. Hayes and her baby farm, and instituted a prosecution in time, starvation and filth would soon have driven her to the grave, to be followed in due course by her little fellow-sufferers, and Mrs. Hayes would have been six pounds the richer by every death. The insurance of child-lives is putting a premium on slow murder. If the murderer—parent or baby-farmer—can produce a certificate of death from a natural cause—pneumonia, typhoid, consumption, any disease—no one on earth can keep from him the price of his crime. The doctor, who might say something about the treatment and conditions that caused the disease, is not permitted to do so; inquests may show a number of moral facts which do not, by some jugglery, form legal evidence, and so the delinquent escapes to try the same safe experiment on some other hapless child. Nothing of any moment can be done till a law is introduced forbidding the insurance of child-lives, except under the most stringent regulations; and till that shall come we hope that Justice Day's words, the expression of a good and honest man's righteous indignation, will ring in the ears of our Parliament and nation.

